# Hypothermic Oxygenated Perfusion Versus Static Cold Storage for Expanded Criteria Donors in Liver and Kidney Transplantation: Protocol for a Single-Center Randomized Controlled Trial

**DOI:** 10.2196/13922

**Published:** 2020-03-19

**Authors:** Matteo Ravaioli, Lorenzo Maroni, Andrea Angeletti, Guido Fallani, Vanessa De Pace, Giuliana Germinario, Federica Odaldi, Valeria Corradetti, Paolo Caraceni, Maurizio Baldassarre, Francesco Vasuri, Antonia D'Errico, Gabriela Sangiorgi, Antonio Siniscalchi, Maria Cristina Morelli, Anna Rossetto, Vito Marco Ranieri, Matteo Cescon, Massimo Del Gaudio, Chiara Zanfi, Valentina Bertuzzo, Giorgia Comai, Gaetano La Manna

**Affiliations:** 1 Department of Medical and Surgical Sciences University of Bologna Sant'Orsola-Malpighi Hospital Bologna Italy; 2 Department of Experimental Diagnostic and Specialty Medicine University of Bologna Sant'Orsola-Malpighi Hospital Bologna Italy; 3 Pathology Division University of Bologna Sant'Orsola-Malpighi Hospital Bologna Italy; 4 Emilia-Romagna Transplantation Referral Center Emilia-Romagna Italy

**Keywords:** organ grafts, organ transplants, perfusion, kidney transplantation, liver transplantation, hypothermia, clinical trials, temperature, randomized

## Abstract

**Background:**

Extended criteria donors (ECD) are widely utilized due to organ shortage, but they may increase the risk of graft dysfunction and poorer outcomes. Hypothermic oxygenated perfusion (HOPE) is a recent organ preservation strategy for marginal kidney and liver grafts, allowing a redirect from anaerobic metabolism to aerobic metabolism under hypothermic conditions and protecting grafts from oxidative species–related damage. These mechanisms may improve graft function and survival.

**Objective:**

With this study, we will evaluate the benefit of end-ischemic HOPE on ECD grafts for livers and kidneys as compared to static cold storage (SCS). The aim of the study is to demonstrate the ability of HOPE to improve graft function and postoperative outcomes of ECD kidney and liver recipients.

**Methods:**

This is an open-label, single-center randomized clinical trial with the aim of comparing HOPE with SCS in ECD kidney and liver transplantation. In the study protocol, which has been approved by the ethics committee, 220 patients (110 liver recipients and 110 kidney recipients) will be enrolled. Livers and kidneys assigned to the HOPE group undergo machine perfusion with cold Belzer solution (4-10°C) and continuous oxygenation (partial pressure of oxygen of 500-600 mm Hg). In the control group, livers and kidneys undergoing SCS are steeped in Celsior solution and stored on ice. Using the same perfusion machine for both liver and kidney grafts, organs are perfused from the start of the back-table procedure until implantation, without increasing the cold ischemia time. For each group, we will evaluate clinical outcomes, graft function tests, histologic findings, perfusate, and the number of allocated organs. Publication of the results is expected to begin in 2021.

**Results:**

Dynamic preservation methods for organs from high-risk donors should improve graft dysfunction after transplantation. To date, we have recruited 108 participants. The study is ongoing, and recruitment of participants will continue until January 2020.

**Conclusions:**

The proposed preservation method should improve ECD graft function and consequently the postoperative patient outcomes.

**Trial Registration:**

ClinicalTrials.gov NCT03837197; https://clinicaltrials.gov/ct2/show/NCT03837197 ; Archived by WebCite® at http://www.webcitation.org/76fSutT3R

**International Registered Report Identifier (IRRID):**

DERR1-10.2196/13922

## Introduction

Transplantation is the ideal therapeutic treatment for end-stage liver and kidney disease. However, this treatment seems to be the victim of its own success. Although the number of liver transplantation (LT) and kidney transplantation (KT) procedures is increasing, as recently reported by the Italian registry, we face a dramatic decrease in the number of available organs [1].

Compared with standard donors, extended criteria donor (ECD) grafts are more vulnerable to the intracellular harmful effects of ischemia, such as a decrease in the availability of adenosine triphosphate (ATP), increase in reactive oxygen species, and release of lysosome enzymes with consequent alteration of cell structure and function [2,3]. In addition, hypoxia inhibits glucose oxidative phosphorylation, leaving anaerobic glycolysis as the only source of ATP production, with consequent alteration of the intracellular ionic environment and phospholipid membrane integrity [2,3]. All these events lead to the severe morphological damage that facilitates the onset of graft dysfunction [2,3].

Organ preservation is crucial when ECD transplant grafts are utilized. To date, static cold storage (SCS) is the most widely used method for organ preservation due to its simplicity and effectiveness in reducing metabolism and the associated oxygen need [4]. However, several studies have reported associations between the SCS preservation of ECD grafts and increased rates of delayed graft function (DGF) and primary graft non-function (PNF) in KT, increased rate of early allograft dysfunction (EAD) in LT, and reduced long-term graft survival [5,6].

Over the last decade, researchers have focused their attention on investigating alternative strategies for organ preservation. Preclinical and clinical studies have explored normothermic (35-37°C), sub-normothermic (20-25°C), and hypothermic (4-10°C) machine perfusion with (hypothermic oxygenated perfusion [HOPE]) or without (hypothermic machine perfusion) oxygen [3,7]. Dynamic perfusion improves the quality of high-risk grafts, removes waste products, and provides metabolic substrates for ATP and glutathione generation, which protects against reactive oxygen species–related damage [7]. Several clinical studies have demonstrated how HOPE improves short-term and long-term outcomes of KT and LT recipients [7-11].

In this study, we will evaluate the benefit of end-ischemic HOPE on ECD grafts (liver and kidney) as compared with SCS. Organs will be perfused through a recently developed machine perfusion device from the beginning of back-table procedures until implantation, without increasing cold ischemia time (CIT). The aim of the study is to demonstrate the ability of HOPE to improve graft function and post-operative outcomes of ECD kidney and liver recipients.

## Methods

### Study Design

In an open-label, single-center, randomized clinical trial, we will compare HOPE (study group) to SCS (control group) in ECD KT and LT. In the HOPE group, 55 livers and 55 kidneys will be preserved by SCS at 4-10°C from the end of organ retrieval until arrival at the transplant hospital. Afterwards, grafts are preserved with HOPE at 4-10°C for a minimum of 1 hour for livers and 2 hours for kidneys until implantation in the recipient. HOPE starts during back-table graft preparation. During the back-table procedure, organs are flushed with a preservation fluid (Belzer solution). Then, the organ is perfused with HOPE through a closed recirculating system.

In the SCS group, 55 livers and 55 kidneys will be preserved by SCS at 4-10°C from the end of the organ retrieval until implantation in the recipient.

In this study, 220 patients will be enrolled, with 55 in each of the following groups: LT-HOPE, LT-SCS, KT-HOPE, and KT-SCS.

**Figure 1:**
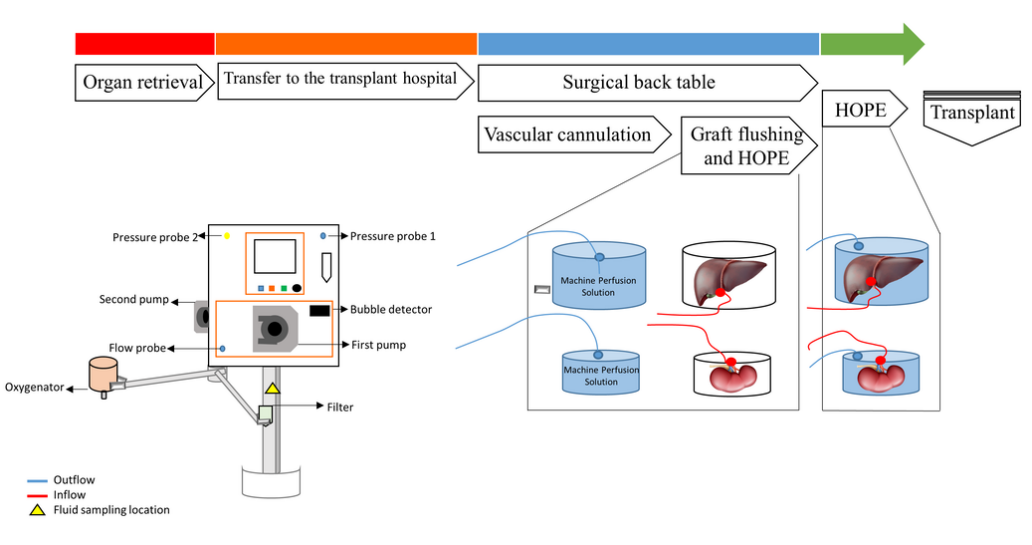
The protocol algorithm for treatment of the study group undergoing preservation with hypothermic oxygenated perfusion (HOPE).

The trial design is outlined in Table 1, and the protocol algorithm is shown in Figure 1.

**Figure 2:**
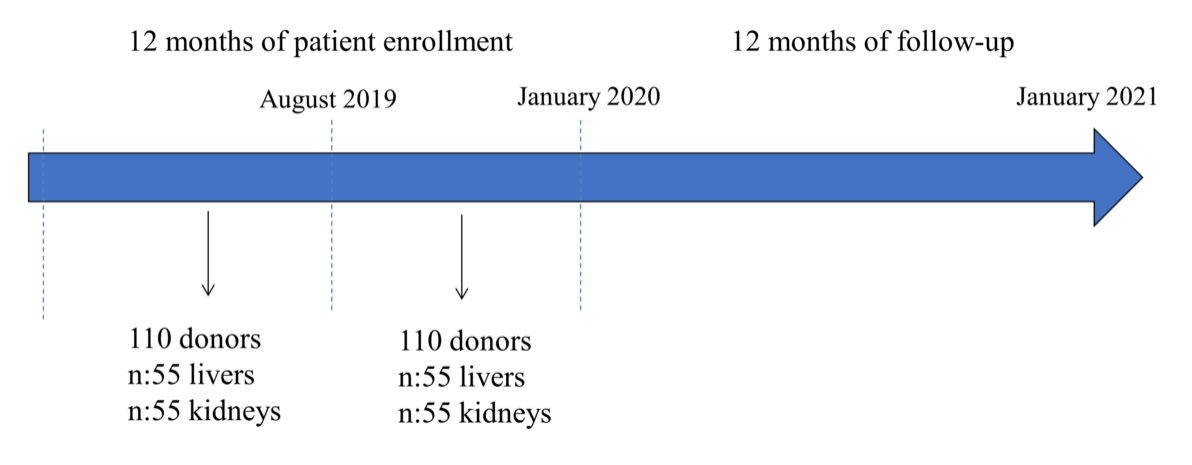
Estimated 2-year timeline to conduct the study, with enrollment at Bologna Transplant center starting in January 2019 and ending in January 2020.

Based on the number of transplants usually performed at the participating center, this clinical study is estimated to be accomplished in 2 years, including 12 months of patient enrollment and 12 months of follow-up. Enrollment at the Bologna Transplant Center started in January 2019 and will end in January 2020 (Figure 2).

**Table 1:** Trial design to evaluate the benefit of end-ischemic hypothermic oxygenated perfusion (HOPE) on extended criteria donor grafts for livers and kidneys as compared with static cold storage (SCS), N=220.

			Study group (HOPE)		Control group (SCS)
Number of livers			55		55
Number of kidneys			55		55
**Process flow**					
	Step 1.		Preservation in SCS at 4-10°C		Preservation in SCS at 4-10°C
	Step 2.		Transfer to the transplant hospital		Implantation in the recipient
	Step 3.		Preservation with flushing and HOPE for 30-40 minutes		N/A
	Step 4.		Preservation with HOPE at 4-10°C for 1-3 hours		N/A
	Step 5.		Implantation in the recipient		N/A

### Study Population

#### Donor and Patient Selection

Donors are considered eligible for the trial if they meet the United Network for Organ Sharing criteria for ECD. For kidneys, these include donor age ≥60 years or 50-59 years plus 2 or more of the subsequent risk factors: death due to cerebrovascular accident, history of hypertension, donor serum creatinine >1.5 mg/dL, or CIT >20 hours. For livers, these include hemodynamic deterioration, donor age >65 years, donor BMI >30 kg/m^2^, serum bilirubin >3 mg/dL, aspartate aminotransferase (AST) or alanine aminotransferase (ALT) >3 times the upper reference threshold, sodium >165 mmol/L, intensive care unit stay >7 days, steatosis >40%, or CIT >12 hours [12,13].

Exclusion criteria include donor age ≤18 years, split-liver recipients, LT for acute liver failure, pre-emptive renal transplant, and intraoperative surgical complications before the organ implantation. Donors after circulatory death will also be excluded, because Italian law requires 20 minutes of a “no touch period” before the death declaration, causing prolonged warm ischemia and subsequent mandatory perfusion of the organ [14].

All adult (age ≥18 years) patients waitlisted for LT or KT will be enrolled in the study after providing written informed consent.

#### Randomization

Patients are randomized 1:1 to the HOPE and SCS groups according to the treatment list produced by the randomizer tool. For KT, patients are stratified according to the duration of CIT before HOPE starts (longer or shorter than 12 hours); grafts with CIT >20 hours are excluded from the study. For LT, patients are stratified according to the contemporary presence of ECD liver criteria (more or less than 5 criteria). The study information and informed consent form are distributed to potential recipients. Randomization is performed for patients who sign the consent form after the organ is deemed suitable for transplantation.

To favor comparison between paired kidneys, when both kidneys from the same donor are allocated to the same center, grafts are automatically assigned to a study group and the corresponding control group. Furthermore, in cases where there are multiple transplants occurring at the same time, we prioritize LT over KT, to reduce the CIT.

### Organ Retrieval

Organs are procured using the technique developed by Starzl. Following aortic clamping, abdominal organs are flushed in situ through the aorta and portal vein with cold Celsior solution, retrieved, dipped in a bag filled with preservation fluid, and stored on ice (1 L, livers; 0.5 L, kidneys). Pretransplant biopsies are performed according to our retrieval protocol.

Retrieved organs are stored on ice during the transfer from donor to the transplant hospital, during the biopsy analysis, until cross-matched results are returned, and until the final decision regarding donor and recipient eligibility.

#### Hypothermic Oxygenated Machine Perfusion

Organ perfusion is conducted with the Vitasmart (Medica, Bologna, Italy) machine, expressly designed for ex vivo perfusion of abdominal organs [7]. This machine system consists of two pumps, one heat exchanger, and three flow and pressure probes (Figure 2). The sterile disposable perfusion set is composed of a membrane oxygenator, tubing for vessel cannulation, and surgical cannulas.

Kidney perfusion is performed through the renal artery at a pressure of 25-30 mm Hg. Liver perfusion is performed through the portal vein at a pressure of 5 mm Hg.

Flow, pressure, and temperature are monitored and stored on a USB memory device during organ perfusion. Gas analysis of the effluent perfusate is accomplished at the start of perfusion (T0) and then every 30 minutes to determine carbon dioxide partial pressure, oxygen partial pressure (pO_2_), pH, and lactate levels. Two perfusate samples are collected at the beginning and at the end of perfusion to rule out bacterial or fungal contamination.

Graft perfusion is performed in the operating room, from the start of the back-table preparation to organ implantation. First, each organ is connected to the perfusion device through cannulation of the vessels with appropriately sized cannulas. HOPE starts by flushing the organ at low flow values (20 mL/min) with new oxygenated perfusion fluid during the back-table preparation, with the aim of removing waste products and residual microthrombi. After the back-table preparation is completed, the organ is treated with continuous HOPE until transplant. Organ perfusion is continuously monitored. As previously reported [7,8], minimal perfusion time is 1 hour for livers and 2 hours for kidneys.

Belzer machine perfusion solution (2 L, kidneys; 3 L, livers) at 4-10°C, in sterile conditions, and with continuous oxygenation (pO_2_ of 500-600 mm Hg) is used for perfusion.

#### Static Cold Storage

Livers/kidneys undergoing SCS are stored in sterile organ bags with Celsior solution and cooled on ice (0.5 L, kidneys; 1 L, livers).

### Transplantation, Immunosuppressive Therapy, and Management During Hospital Stay

KT and LT are performed according to the center’s standard techniques. Kidneys are implanted into either the iliac fossa with arterial anastomoses to the external, common, or internal iliac arteries or vein anastomoses to the external or common iliac veins and ureter-bladder anastomoses over a single stent. Livers are transplanted orthotopically preserving the inferior vena cava with a piggyback technique.

Postoperative management, including immunosuppression and antimicrobial, antifungal, and antithrombotic prophylaxis, follows the standard local protocol [15].

### Analyses of Outcomes

#### Liver Transplant Primary Outcome

**Figure 3:**
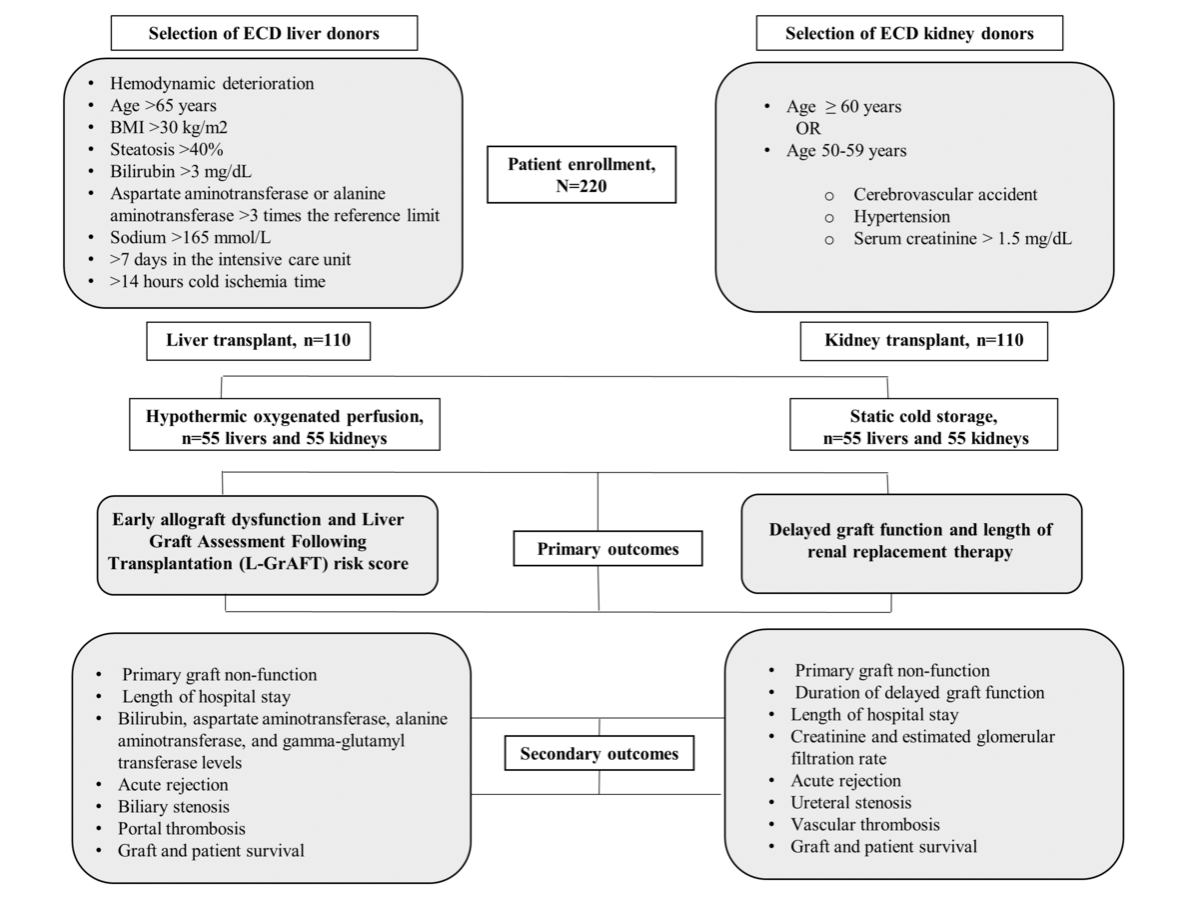
Inclusion criteria and postoperative outcomes of enrolled extended criteria donor (ECD) liver and kidney recipients.

The rate of EAD and the Liver Graft Assessment Following Transplantation (L-GrAFT) risk score [16,17] will be analyzed to evaluate the postoperative outcomes of the enrolled liver recipients (Figure 3).

EAD is defined by the presence of at least one of the following lab results: bilirubin >10 mg/dL, international normalized ratio >1.6 on postoperative day 7, ALT >2,000 IU/mL within the first 7 postoperative days, or AST >2,000 IU/mL within the first 7 postoperative days [17]. The L-GrAFT risk score is calculated from the peak AST level, bilirubin levels, platelet counts, and international normalized ratio values from days 1 to 10 post-LT [17].

#### Kidney Transplant Primary Outcome

The incidence and timing of DGF will be evaluated and correlated with the outcomes of the kidney recipients (Figure 3). DGF is defined as the need for renal replacement therapy during the first week posttransplant [18]. The length of renal replacement therapy is calculated as the interval between the first day and last day of dialysis.

#### Liver Transplant Secondary Outcomes

Secondary outcome measures in the LT groups are the incidence of PNF, defined as patient death or the need for early retransplantation within the first 7 postoperative days, excluding acute vascular complications [18]; postreperfusion syndrome rate; length of hospital stay (LHS); liver function test values (ie, bilirubin, AST, ALT, and gamma glutamyl transferase) at discharge and 1, 3, and 6 months after LT; occurrence of acute rejection events, biliary complications, portal vein thrombosis, or hepatic artery thrombosis within 6 months from the transplant; graft survival, defined as the time from transplant to retransplant or patient death due to liver failure; and patient survival.

#### Kidney Transplant Secondary Outcomes

Secondary outcomes for kidney recipients are the incidence of PNF, defined as irreversible graft dysfunction with graft loss, which can also be due to rejection or vascular thrombosis; postreperfusion syndrome rate; number of dialyses required in the posttransplant follow-up; LHS; renal function, as measured using creatinine level and estimated glomerular filtration rate, at discharge, 1, 3, and 6 months posttransplant; occurrence of acute rejection events, anastomotic or nonanastomotic ureteral stenosis, or vascular thrombosis within 6 months from the transplant; graft survival, defined as the time from transplant to graft loss or return to dialysis; and patient survival.

### Biomarkers of Oxidative Stress, Inflammation, and Ischemia-Reperfusion Injury

Tissue and perfusate samples are assessed for oxidative stress and metabolic state using reverse transcription polymerase chain reaction (RT-PCR), enzyme-linked immunosorbent assay (ELISA), Luminex technology multiplex assays, and mass spectroscopy analysis. Tissue and perfusate samples are also assessed for the inflammatory markers CD39, CD73, E-selectin, vascular cell adhesion molecule 1, intercellular adhesion molecule 1, hypoxia-inducible factors to tumor necrosis factor-α, interleukin 6, and interleukin 8 using Luminex technology multiplex assays and/or ELISA and RT-PCR.

Specific markers related to renal function and tubular injury, including alpha-1-microglobulin, beta-2-microglobulin, albumin, clusterin, cystatin C, epidermal growth factor, neutrophil gelatinase-associated lipocalin, osteopontin, urinary total protein, trefoil factor 3, hepatocyte growth factor, and macrophage stimulating protein, are measured using Luminex technology multiplex assays and/or ELISA in perfusate and/or ultrafiltrate samples collected before and after ex vivo organ reperfusion.

Adenosine, ATP, adenosine diphosphate, adenosine monophosphate, riboflavin, and succinate levels are measured in tissue, perfusate, ultrafiltrate, and reperfusion fluid using high-performance liquid chromatography.

Finally, miR-21, a small target molecule involved in renal and liver ischemia-reperfusion injury processes, is measured.

### Tissue and Vascular Morphology

Tissue injury is evaluated using histopathological analysis before and after ex vivo organ perfusion. Biopsy samples are taken during organ retrieval to assess graft suitability, after graft reperfusion into the recipient, and at the end of the transplant. Pretransplant biopsies are obtained according to our center practice. All tissue samples are sent to Bologna Transplant Center to reduce interlaboratory bias in preparing the slides.

For the livers, parenchymal and vascular damage are evaluated. For the kidneys, glomerular, vascular, tubular, and parenchymal damage are evaluated. Tissue samples are examined by two double-blind pathologists, who grade any damage from moderate to severe.

In addition, we perform immunohistochemical assays to investigate endothelial and epithelial cell injury, ischemia- reperfusion damage, the pro-inflammatory and anti- inflammatory environment, growth factors, dedifferentiation, repair, and apoptosis using specific stainings. Electron microscopy is performed to assess the features of oxidative injury, focusing on preservation of epithelial mitochondria, endothelial cells of the glomerular and peritubular capillaries, and liver sinusoids.

Tissue and perfusate samples are collected and coded to guarantee privacy and data protection in accordance with EU regulations. Perfusate and tissues samples collected for RT-PCR are snap-frozen and stored at –80°C. Tissue collected for immunohistochemical assays are preserved in glutaraldehyde solution.

### Posttransplant Follow-up

Postoperative follow-up is carried out according to the local protocol. Hepatic and renal function tests and abdominal ultrasound are performed at each follow-up visit. Follow-up will end at 12 months.

### Risk Analyses

Technical assistance by a team of expert technicians is provided during all phases of the machine perfusion procedures.

Vascular endothelial damage is avoided by perfusing the liver solely through the portal vein, leaving the hepatic artery untouched, and keeping the perfusion pressure of the renal artery and portal vein at low levels, which we previously demonstrated as being safe in preclinical and clinical studies [7,8].

### Data Management

Demographic, clinical, and biological data of donors and recipients are collected and prospectively entered in the database. The data registration is anonymous, and a study identification code is assigned to each transplant, in accordance with the Helsinki Declaration.

Missing values are handled properly by the researchers to achieve accurate inferences about the data during the analyses of the results.

### Statistical Analyses

#### Sample Size Calculation

The sample size was calculated using the primary outcomes of EAD and DGF for LT and KT, respectively, and the secondary outcomes of PNF and LHS, as reported in similar studies [11,19,20].

In particular, the rates of PNF and EAD are expected to decrease for the LT-HOPE group, thereby improving the postoperative course and LHS. We are expecting the following reductions in these parameters: PNF, 5% (HOPE) vs 10% (SCS); EAD, 10% (HOPE) vs 20% (SCS); and postoperative LHS <21 days, 80% (HOPE) vs 50% (SCS).

In KT, HOPE should reduce the rate of DGF from 50% to 30%, with a general improvement in the postintervention outcomes.

Based on these parameters, the total sample size was estimated at 220 patients, with distribution throughout the 4 groups as already explained (alpha=.05, two-sided test, power of 80%; calculated with nQuery Advisor 7.0, Statsols, Cork, Ireland).

The sample size was calculated to account for a dropout risk of 5-10% for the year.

A preliminary analysis will be performed when half of the enrollment is completed.

#### Preliminary Analyses

To monitor and optimize the planned surgical and clinical procedures, real-time data analyses are performed before the final data collection. In detail, we are performing two interim analyses on the data from the first 7 posttransplant days. The primary analyses will be performed after the enrollment of 60 patients. The second analyses will be performed after the enrollment of 160 patients.

#### Statistical Tests

The continuous variables will be compared using parametric (ANOVA) or non-parametric (Kruskal-Wallis) tests according to the data distribution, and the categorical variables will be compared using chi-squared tests. Multivariate analysis will be performed using forward stepwise logistic regression analysis. Survival analysis will be conducted using the Kaplan-Meier method. *P* values <.05 will be considered statistically significant.

### Ethical Review

The research protocol, including the forms for data treatment, study information, and participant content forms, were approved by the Emilia-Romagna Region Ethics Committee, which is the ethics committee for the transplant center, and the National Health System Research.

Written, informed consent is obtained prior to final enrollment. Randomization is performed as recommended by the ethics committee, and all members of the research team learns the treatment type of each recipient only after their inclusion. Possible protocol changes will be applied after the approval of their amendments by the National Health System Research and then the local ethics committee.

Recipients are insured against study-related adverse events with a protocol-specific insurance policy.

The principal investigator and all members of our research scientific group have declared no conflict of interest.

### Dissemination

We will describe the data and transcribe the results with the aim to develop an original article for submission at a scientific review, national conferences, and international conferences.

### Clinical Relevance

With ECD LT and KT, the use of adequate organ preservation techniques may improve posttransplant outcomes without compromising graft function and survival, thereby increasing the donor organ pool.

## Results

Dynamic preservation methods for organs from high-risk donors should improve the functional recovery of the graft with a lower expected DGF for KT and EAD for LT.

To date, we have recruited 108 participants. The study is ongoing, and recruitment of participants will continue until January 2020.

## Discussion

This study suggests that the use of adequate organ preservation techniques may improve the posttransplant outcomes without compromising graft function and survival, thereby increasing the donor organ pool. The concept of dynamic organ preservation was developed by Carrel and Lindbergh in the 1930s [21,22]. An increasing number of ECDs are used for transplantation, which has triggered an interest in new preservation techniques to improve organ quality and decrease the occurrence of severe complications [23,24]. While the use of oxygen in machine perfusion for liver preservation has been extensively investigated in clinical trials, HOPE has been reported less frequently for KT [25].

An important aspect of this study is the use of the same perfusion device for liver and kidney grafts, which differs from previous studies [11,20,26,27]. With this machine, organ perfusion is a simple procedure that can be started during the back-table preparation of the surgical graft, avoiding an increase in CIT.

Increasing evidence suggests that HOPE of the graft should start immediately after retrieval. This might reduce the accumulation of waste products, such as succinates, and the perfusion should result in better restoration of mitochondrial function [10-25].

Our protocol starts with washing the graft for 30-40 minutes during organ preparation to completely remove the waste elements that were released and accumulated from the start of ischemia. And, the organ perfusion system is equipped with an adsorbing hemofilter to remove cytokines and avoid fat embolism. Following this step, oxygenation and recirculation of the preservation fluid begin. Flow, pressure, and temperature are monitored and stored on a USB memory device during organ perfusion. Gas analysis of the effluent perfusate is conducted at the start of perfusion (T0) and then every 30 minutes to determine carbon dioxide partial pressure, pO_2_, pH, and lactate levels. Two perfusate samples are collected at the beginning and at the end of perfusion to rule out bacterial or fungal contamination.

Another important aspect is the simplicity of this organ preservation procedure in terms of organization and management. We do not need a perfusion specialist, and we have had no procedure-related adverse events. In addition, starting from the back-table procedures, CIT is not prolonged.

Advantages of this HOPE system include its simplicity and improved LT and KT outcomes.

In conclusion, we aim to demonstrate the ability of HOPE to improve graft function and postoperative outcomes of ECD kidney and liver recipients.

## Appendix

Multimedia Appendix 1 CONSORT-eHEALTH checklist (V 1.6.1).

## Abbreviations

ALT: alanine aminotransferase.
AST: aspartate aminotransferase.
ATP: adenosine triphosphate.
CIT: cold ischemia time.
DGF: delayed graft function.
EAD: early allograft dysfunction.
ECD: extended criteria donor.
ELISA: enzyme-linked immunosorbent assay.
HOPE: hypothermic oxygenated perfusion.
KT: kidney transplantation.
L-GrAFT: Liver Graft Assessment Following Transplantation.
LHS: length of hospital stay.
LT: liver transplantation.
PNF: primary graft non-function.
RT-PCR: reverse transcription polymerase chain reaction.
SCS: static cold storage.
